# Kombucha tea as an anti-hyperglycemic agent in humans with diabetes – a randomized controlled pilot investigation

**DOI:** 10.3389/fnut.2023.1190248

**Published:** 2023-08-01

**Authors:** Chagai Mendelson, Sabrina Sparkes, Daniel J. Merenstein, Chloe Christensen, Varun Sharma, Sameer Desale, Jennifer M. Auchtung, Car Reen Kok, Heather E. Hallen-Adams, Robert Hutkins

**Affiliations:** ^1^Department of Medicine, MedStar Georgetown University Hospital, Washington, DC, United States; ^2^Department of Human Science, Georgetown University School of Health, Washington, DC, United States; ^3^Department of Family Medicine, Georgetown University Medical Center, Washington, DC, United States; ^4^Department of Food Science and Technology, University of Nebraska, Lincoln, NE, United States; ^5^Division of General Internal Medicine, MedStar Georgetown University Hospital, Washington, DC, United States; ^6^MedStar Health, Washington, DC, United States

**Keywords:** kombucha, microorganisms, diabetes mellitus, human subjects, clinical trial

## Abstract

**Introduction:**

Kombucha is a popular fermented tea that has attracted considerable attention due, in part, to its suggested health benefits. Previous results from animal models led us to hypothesize kombucha may reduce blood sugar levels in humans with diabetes. The objective of this pilot clinical study was to evaluate kombucha for its anti-hyperglycemic activities in adults with diabetes mellitus type II.

**Methods:**

The study was organized as a prospective randomized double-blinded crossover study at a single-center urban hospital system. Participants (*n* = 12) were instructed to consume either a kombucha product or a placebo control (each 240 mL) for 4 weeks. After an 8-week washout period, participants consumed the alternate product. Fasting blood glucose levels were self-determined at baseline and at 1 and 4 weeks during each treatment period. Secondary health outcomes, including overall health, insulin requirement, gut health, skin health, mental health, and vulvovaginal health were measured by questionnaire at the same time points. The kombucha microbiota was assessed by selective culturing and 16S rRNA gene (bacteria) and ITS (fungi) sequencing. Fermentation end products were assessed by HPLC. Statistical significance of changes in fasting blood glucose was determined using paired, two-tailed student’s *t*-tests.

**Results:**

Kombucha lowered average fasting blood glucose levels at 4 weeks compared to baseline (164 vs. 116 mg/dL, *p* = 0.035), whereas the placebo did not (162 vs. 141 mg/dL, *p* = 0.078). The kombucha microbiota, as assessed by cultural enumeration, was mainly comprised of lactic acid bacteria, acetic acid bacteria, and yeast, with each group present at about 10^6^ colony forming units (CFU)/mL. Likewise, 16S rRNA gene sequencing confirmed that lactic acid and acetic acid bacteria were the most abundant bacteria, and ITS sequencing showed Dekkera was the most abundant yeast. The primary fermentation end products were lactic and acetic acids, both less than 1%. Ethanol was present at 1.5%.

**Discussion:**

Although this pilot study was limited by a small sample size, kombucha was associated with reduced blood glucose levels in humans with diabetes. Larger follow-up studies are warranted.

**Clinical trial registration:**

ClinicalTrials.gov, identifier NCT04107207.

## Introduction

Diabetes mellitus is the ninth leading cause of death in the world (1). It is also a major risk factor associated with several health complications, including coronary heart disease, stroke, peripheral vascular disease, kidney failure, and an overall decreased quality of life (2, 3). Rates of diabetes have increased more than 400% in the past 30 years, mostly as type 2 diabetes (T2D) (4). The near 15% prevalence rate of diabetes in the U.S. (5), as well as increased rates of diabetes in low- and middle-income countries throughout the world (6), has led to a determined search for dietary approaches that may reduce the burden of this disease (1, 7, 8). In particular, diets rich in fermented foods have been reported to be associated with reduced risk of T2D (9–11).

Kombucha is a beverage obtained by fermentation of sweetened tea with a symbiotic consortium of bacteria and yeasts (SCOBY). It has been consumed in many parts of the world for centuries, both for its taste and perceived beneficial effects on human health (12, 13). In the past decade, kombucha has become especially popular, with a global market of nearly 1.7 billion dollars in 2019 and predicted annual growth of 20% (14, 15). Historically, kombucha has been suggested to have multiple potential health benefits, including reducing risks of cardiovascular disease, cancer, bacterial and viral infections, anxiety and depression, and diabetes (13, 16). These suggested benefits could be mediated by organic acids or other fermentation end-products, by tea consituents, or by added flavoring ingredients, such as ginger, fruit, mint, and herbs that have potential bioactive properites (17, 18). However, despite the substantial consumer interest and these suggested health benefits of kombucha, we have found only a single, recently published work describing results of a clinical trial assessing health benefits of kombucha consumption in human subjects. In this study, the authors demonstrated consumption of kombucha along with a carbohydrate-rich meal significantly reduced increases in plasma glucose and insulin compared to placebo treatment in healthy adults (19).

In contrast, several studies have shown various metabolic benefits of kombucha in animal models. Aloulou et al. demonstrated the anti-hyperglycemic effects of kombucha compared to non-fermented black tea in diabetic rats, with histological evidence of pancreatic beta cell regeneration (20). Srihari et al. (21) reported significant decreases in glycosylated hemoglobin (HbA1c) levels in diabetic rats after 45 days of kombucha consumption, and Zubaidah et al. (22) showed a significant reduction of fasting plasma glucose in diabetic rats after 28 days, also with enhanced beta cell regeneration. More recently, kombucha tea was shown to improve glycemic parameters, including glucose tolerance, and attenuate symptoms associated with nonalcoholic fatty liver disease in obese mice (23), to provide protective effects against lipopolysaccharide (LPS)-induced sepsis in LPS-challenged mice (24), and to reduce hyperglycemia in T2D-induced mice (25). In general, these effects were mediated by changes in the gut microbiota and their metabolic end-products.

Based on results from animal studies, we hypothesized kombucha could decrease blood sugar levels in human diabetic subjects. Therefore, in this pilot study, we tested kombucha as an anti-hyperglycemic therapeutic agent in human adult participants with T2D. We observed that kombucha consumption by subjects with elevated blood glucose levels at baseline was associated with a significant reduction in fasting blood glucose that was not observed during consumption of a placebo beverage. To our knowledge, our study is the first randomized controlled human trial in which the anti-diabetic effects of kombucha were assessed among diabetic participants.

## Materials and methods

### Overall study design

This was a pilot, single-center randomized, double blinded, cross-over study performed at Georgetown University Medical Center in Washington, DC. Recruitment of participants was performed at MedStar Georgetown University Hospital’s General Internal Medicine Clinic, a primary care clinic with a strong interest in diabetes care. Participants were recruited during October–November 2019, interventions were given in December 2019 through February 2020. This study was approved by the Institutional Review Board committee at MedStar and performed in accordance with their guidance (IRB ID STUDY00001101). The trial was registered at ClinicalTrials.gov: Identifier: NCT04107207 on September 27, 2019.

### Selection and enrollment of participants

**Figure 1:**
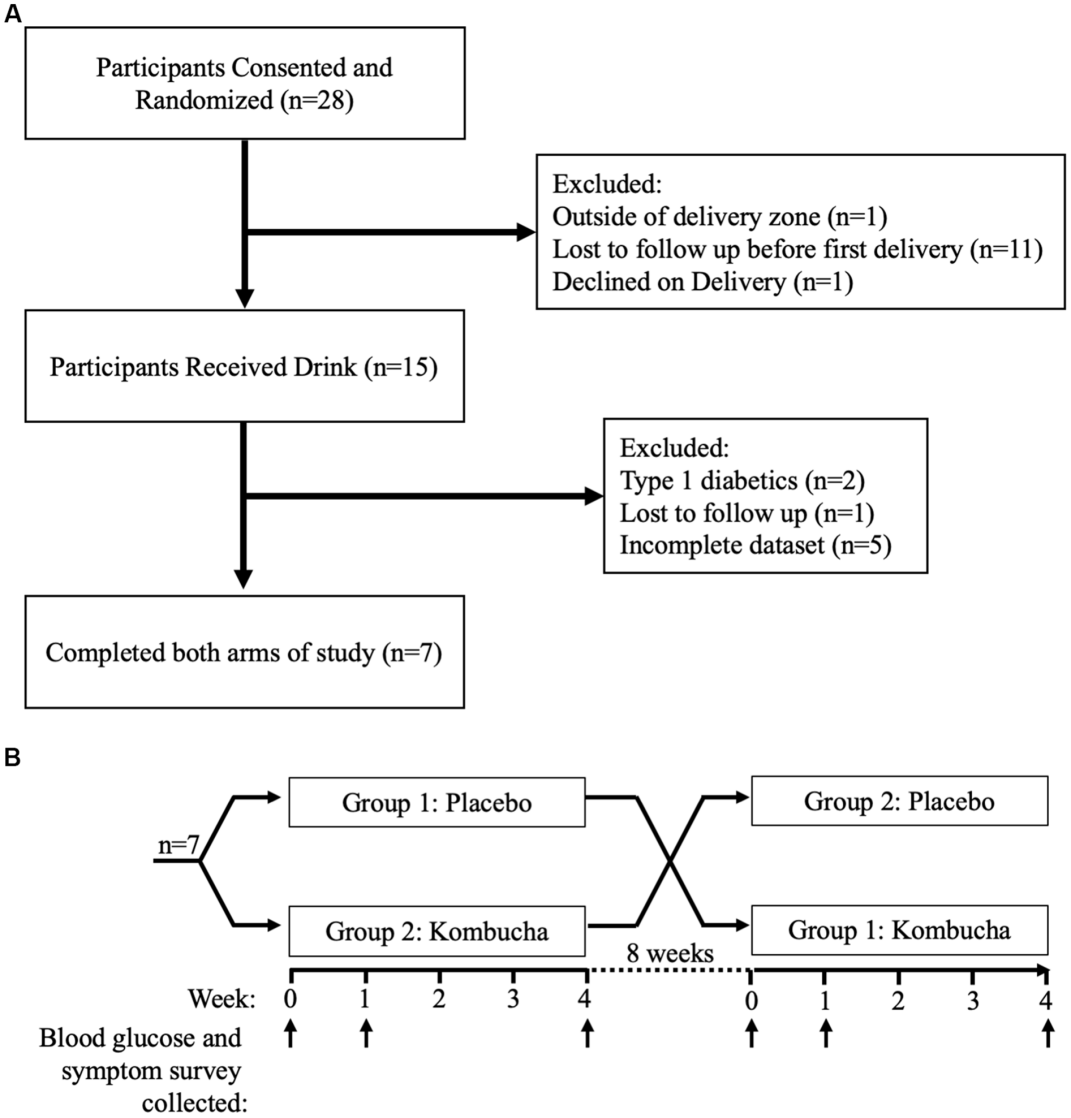
**(A)** Study population identification and exclusion process. **(B)** Crossover study design and sample collection.

Participants were recruited using enrollment posters posted in the clinic. Inclusion criteria included: diagnosis of T2D and willingness to check daily blood glucose levels, age of 18 years or above, ability to read, speak and write English, and telephone access. Exclusion criteria included: allergy to kombucha ingredients or regular kombucha consumption. Randomization was performed in blocks of 2 using the RAND function of Microsoft Excel. Study population and exclusion criteria are outlined in Figure 1A.

### Study interventions

Participants were instructed to consume 240 mL (8 ounces) of a study drink with dinner, daily, for 4 weeks. The study drink was either kombucha or a placebo drink. After a washout period of 8 weeks, participants then consumed 240 mL of the other study drink, also daily for 4 weeks (Figure 1B). Researchers and participants were blinded to the type of drink. The kombucha was made using traditional fermentation with a SCOBY starter culture, followed by a secondary fermentation in kegs. The kombucha was produced specifically for this study by Craft Kombucha, a commercial kombucha manufacturer in the Washington, DC area. The placebo drink was an unfermented sparkling drink, also prepared by Craft Kombucha specifically for this study and intended to be similar to the kombucha in flavor and appearance. The placebo was made from heat-treated ingredients, cooled, diluted with water, and carbonated. Both the kombucha and placebo were sweetened with sucrose (10%, w/w) and flavored with the same amount of freeze-dried ginger powder (0.6%, w/w) to match flavor profiles. Ginger flavoring was chosen because it is among the most common flavors in the commercial kombucha market (14, 26), and also it is an ingredient in the producer’s basic kombucha formulation.

### Data collection

Participants reported daily fasting blood glucose levels (measured using a home fingerstick testing glucose meter before eating breakfast) on a weekly basis at six timepoints: at baseline, at the end of 1 week of the first drink, at the end of 4 weeks of the first drink, at baseline after a washout period, at the end of 1 week of the second drink, and at the end of 4 weeks of the second drink. Morning fasting blood glucose levels was selected for pragmatic reasons, as this is the most common time in which people with diabetes check their sugars. Only participants who completed both arms and reported blood glucose levels for all six timepoints were included in the analyzes. Self-reported health outcomes were collected at the same six timepoints.

### Outcomes

The primary outcome was the average fasting blood glucose levels as reported by participants. Secondary outcomes were self-reported health outcomes on a health questionnaire (Supplementary Files S1–S3) and included overall health, insulin requirement, gut health and related symptoms, skin health and related symptoms, mental health and related symptoms, and vulvovaginal health and related symptoms. All outcomes were listed *a priori* on clinicaltrials.gov. Microbiota and chemical analyzes of the kombucha were also performed.

### Questionnaire design

The questionnaire (Supplementary File S1) was adapted for this study, based on previously validated questionnaires. For gut health questions, the survey was adapted from the Rome IBS questionnaire (27), for mental health questions it was adapted from the Generalized Anxiety Disorder (28) and the PHQ2 (29), and for vulvovaginal health questions it was adapted from a previously published questionnaire (30).

### Microbial analysis

Kombucha liquid samples were sent to the University of Nebraska-Lincoln by overnight delivery for microbiological analysis. The samples were packed with freezer packs and were cold upon receipt. Aliquots (1 mL) were removed and stored at −20°C in 1.5 mL microcentrifuge tubes for DNA extraction. To enumerate microorganisms by cultural methods, the fresh samples were mixed and serially diluted with saline (0.9% NaCl) solution. For total aerobic bacteria, samples were plated on plate count agar (PCA) and incubated aerobically for 48 h at 30°C. For lactic acid bacteria (LAB), MRS agar (BD Difco) was used, with anaerobic incubation for 48 h at 30°C. For acetic acid bacteria (AAB), dilutions were plated onto Wallerstein Laboratory nutrient agar (WL; Sigma-Aldrich) and incubated aerobically for 48 h at 30°C. Yeasts were enumerated on CHROMagar Candida (CHROMagar) after incubation at 30°C for 1 week. Finally, filamentous fungi samples were plated on Sabouraud media and incubated at 30°C for 1 week.

For community-wide analysis, frozen samples were thawed, and DNA was extracted by the phenol-chloroform method as described by Martinez et al. (31), with the exception that the DNA pellets were resuspended in DNase-free water. The DNA was concentrated using ethanol precipitation, and the 16S rRNA gene and ITS regions, for bacteria and fungi, respectively, were amplified and sequenced, and analyzed by Novogene Corporation Inc. (Sacramento, CA). Briefly, Operational Taxonomic Units (OTUs) were generated with UPARSE at 97% sequence similarity (32). Taxonomic identification of representative sequences was performed using the SSUrRNA SILVA (33) and the UNITE databases (34) for 16S rRNA and ITS sequences, respectively. OTUs that were present at less than 0.001% relative abundance were removed and Metacoder was used to visualize microbiota taxonomy and abundance (35).

### Chemical analysis

The principal organic acids found in kombucha were determined by high performance liquid chromatography (HPLC) as described by Lui and Wilkins (36). Briefly, kombucha and the unfermented placebo tea were centrifuged to remove bacteria, supernatant was filtered (0.45 μm), and 10 μL of filtrate was injected into an Aminex HPX-87H column. The column temperature was 65°C, and 5 mM sulfuric acid was used as the mobile phase with a flow rate of 0.6 mL/min. Organic acids were measured using a UV detector. The ethanol concentration was determined using a refractive index detector (RID) at 50°C. Serial dilutions of known quantities of each organic acid and ethanol were also analyzed. The peak area of each sample and standard was calculated by the LC Open Lab program, with analyte concentrations for each sample determined through comparison to standard curves.

### Data collection

Study data were collected and managed using REDCap (37, 38). REDCap (Research Electronic Data Capture) is a secure, web-based software platform designed to support data capture for research studies, providing 1) an intuitive interface for validated data capture; 2) audit trails for tracking data manipulation and export procedures; 3) automated export procedures for seamless data downloads to common statistical packages; and 4) procedures for data integration and interoperability with external sources.

### Statistical analysis

To reduce day-to-day variation in values, seven day mean blood glucose values were calculated for each time point. Paired T-tests were used to compared mean blood glucose levels across subjects from baseline to treatement week 4 and between treatments at week 4. Statistical analysis was not performed on secondary outcome measures.

### Availability of data and materials

The 16S rRNA and ITS sequencing datasets supporting the conclusions of this article are available in the Sequence Read Archive (SRA) repository, with accession number PRJNA928483 at https://www.ncbi.nlm.nih.gov/bioproject/928483.

## Results

### Study population

Twenty-eight participants responded to our recruitment efforts and were consented by phone and then randomized. Of these, eleven participants withdrew before the first drink was delivered. Of the remaining seventeen, one participant lived outside of the delivery zone, and another participant declined the drink at time of delivery. Fifteen participants received the first drink, including one who withdrew and two type-1 diabetics that were excluded from analysis post-hoc. In total, twelve participants were included for analysis, although five had incomplete data sets. Therefore, while the symptoms questionnaire included data for all twelve participants, only the seven participants (Figure 1A) who completed the entire study were included for analysis of fasting blood glucose.

### Participant population

The mean age of the twelve participants was 57 years (range 40–71, SD 8), with half of all participants in their 50s (Table 1). Nine participants (75%) were female. Half were African American and half were white. Two (17%) were current smokers, four (33%) were former smokers, and six (50%) never smoked. Nine participants (75%) were on insulin therapy.

**Table 1:** Participant characteristics.

Gender	*N* (%)	Race	*N* (%)	Insulin use	*N* (%)
Female	9 (75%)	White	6 (50%)	Yes	9 (75%)
Male	3 (25%)	African American	6 (50%)	No	3 (25%)

Age group	*N* (%)	Smoking Status	*N* (%)
40–49	2 (17%)	Never	6 (50%)
50–59	6 (50%)	Former	4 (33%)
60+	4 (33%)	Current	2 (17%)

### Primary outcome

**Figure 2:**
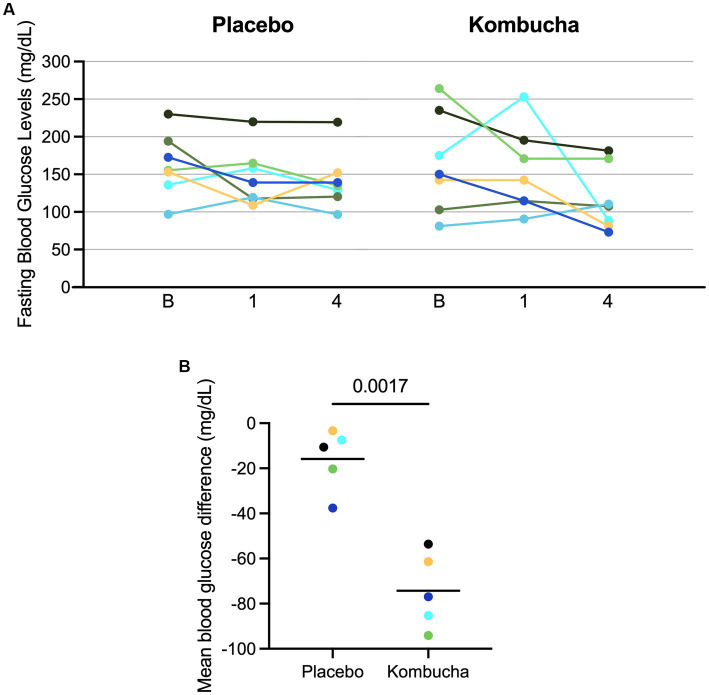
Blood sugar levels for individual participants receiving kombucha or placebo. **(A)** 7-day mean fasting blood sugar levels for subjects collected at baseline (B), week 1 (1), and week 4 (4) for each treatment. Results from each patient are color-coded across each study arm. **(B)** Difference from baseline values in fasting blood glucose after 4 weeks of treatment for five subjects with elevated blood glucose (>130 mg/dL) at baseline.

Overall, Kombucha significantly lowered average fasting blood glucose levels at week 4 compared to baseline (164 vs. 116 mg/dL, *p* = 0.035) (Table 2; Figure 2). The placebo was not associated with a statistically significant change in average fasting blood glucose levels (162 vs. 141 mg/dL, *p* = 0.078). However, despite the significant difference between baseline and week 4 for the kombucha treatment, the difference between fasting blood glucose levels at 4 weeks between treatment and placebo was not statistically significant (*p* = 0.265). This overall analysis included two participants whose blood glucose levels at baseline in the Kombucha-treatment arm of the study were less than 110 mg/dL (Figure 2A) and therefore within the range of fasting blood glucose targets for non-pregnant adults with diabetes [80–130 mg/dL, (39)]. If analysis was restricted to the subset of participants with fasting blood glucose levels above this target range at baseline in both treatment arms (>130 mg/dL, *n* = 5 participants), kombucha consumption was associated with a mean decrease of 74.3 ± 16.7 mg/dL of fasting blood glucose levels between week 4 and baseline, which was significantly different (*p* = 0.017) from the mean decrease of 15.9 ± 13.6 mg/dL from baseline to week 4 in participants consuming the placebo drink. Likewise, the difference between placebo and kombucha treatment was also significant (*p* = 0.0013) when only those five participants were included in the analysis (Figure 2B).

**Table 2:** Seven day mean fasting blood glucose levels at baseline and week 4.

	Fasting blood glucose levels (in mg/dL)^a^				p-value^c^
	Baseline		Week 4		
	Mean	SD^b^	Mean	SD^b^	
Placebo	162.6	42.4	141.7	38.4	0.078
Kombucha	164.3	66.4	116.1	43.2	0.035

a Only subjects that had baseline and week 4 data for both arms of the study were used for statistical analysis (*n* = 7).

b Standard deviations (SD) are also shown.

c Two-tailed, paired student’s T-test was used to calculate *p*-values.

### Secondary outcomes

Because the majority of survey respondents indicated that their symptoms were average or better at all time points analyzed during both treatment arms and not all subjects scored all symptoms at each time point which led to vary small sample sizes (Supplementary Files S2, S3), no further statistical analysis were performed on secondary outcomes.

### Microbiological analysis of kombucha

Based on cultural enumeration, the kombucha contained mostly lactic acid bacteria (1.1 × 10^6^ CFU/mL), acetic acid bacteria (9.5 × 10^5^ CFU/mL), and yeast (7.5 × 10^5^ CFU/mL). Molds were present at about 2.0 × 10^2^/mL. Microbes in the placebo were below detection (10^2^ CFU/mL).

**Figure 3:**
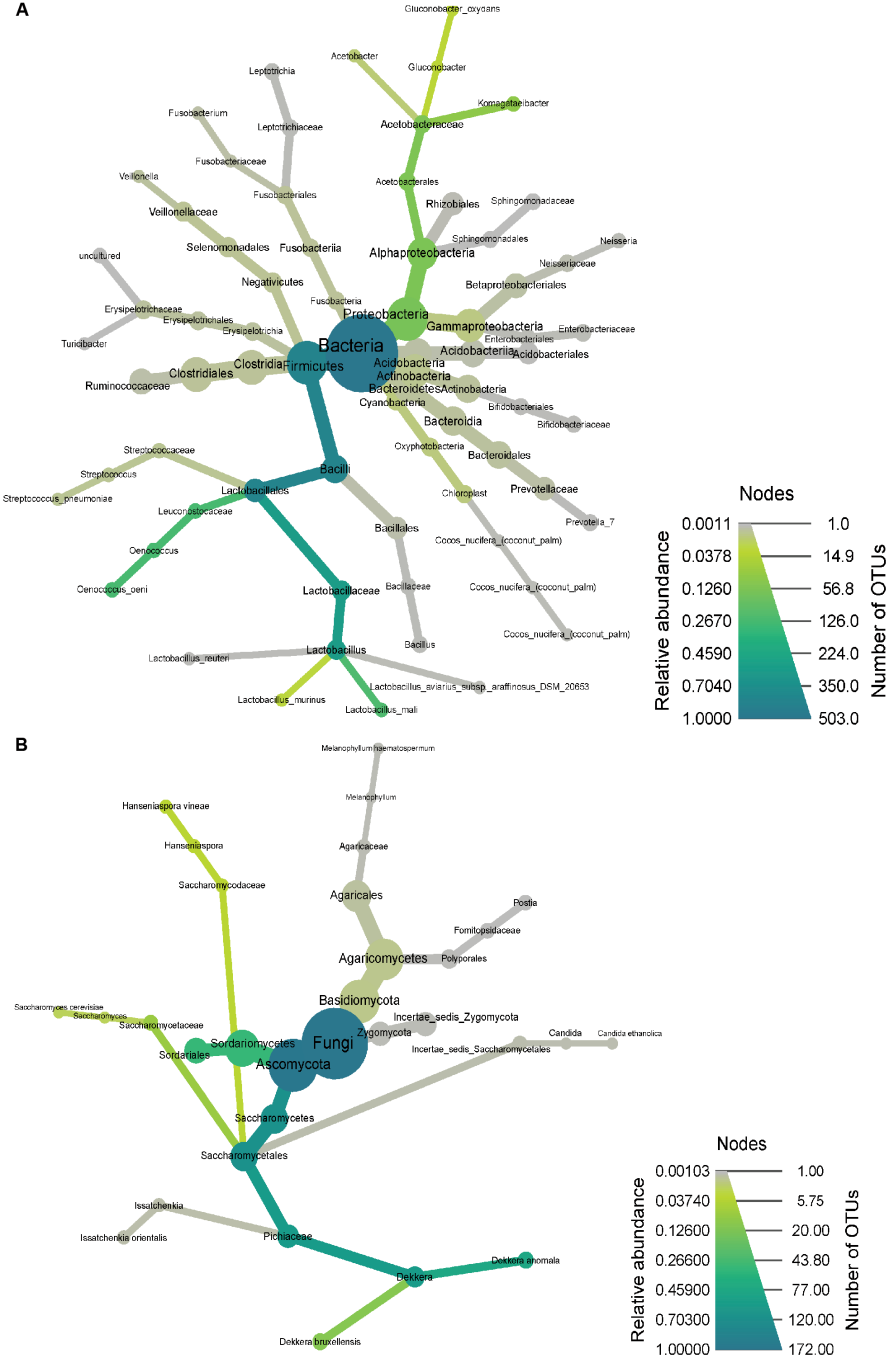
Community microbiota observed through 16S rRNA gene sequencing for bacteria **(A)** and ITS sequencing for fungi **(B)**. Heat trees were generated using Metacoder, where node size and color reflect the number of OTUs that correspond to a particular taxa and taxonomic relative abundance, respectively.

The relative abundances of the primary groups of bacteria and fungi, were also determined by 16S rRNA gene and ITS sequencing, respectively (Figure 3). Among the bacteria (Figure 3A), the kombucha community consisted mainly (>80%) of Bacillota (formerly Firmicutes), with several genera within the family Lactobacillaceae as the predominant members. Included were several genera formerly classified as *Lactobacillus* (40), as well as *Oenococcus* (55 and 26%, respectively). The other major phylum, at 12% abundance, was Pseudomonadota (formerly Proteobacteria), which consisted of several genera of acetic acid bacteria, including *Komagataeibacter*, *Gluconobacter*, and *Acetobacter*.

Presumptive species level assignments (based on 97% identity) revealed that the Lactobacillaceae consisted of *Liquorilactobacillus mali* (formerly *Lactobacillus mali*), *Ligilactobacillus murinus* (formerly *Lactobacillus murinus*), *Limosilactobacillus reuteri* (formerly *Lactobacillus reuteri*), *Ligilactobacillus aviarius* (formerely *Lactobacillus aviarius*), *Lactobacillus gasseri*, and *Ligilactobacillus salivarius* (formerly *Lactobacillus salivarius*). Other species included *Streptococcus pneumoniae* and *Streptococcus variani*, as well as the acetic acid bacterium, *Gluconobacter oxydans*.

Of the yeast and fungi determined by ITS sequencing (Figure 3B), the kombucha was composed mainly of *Dekkera* and *Sordariales*, respectively. Species (97% identity) included *Calycina discreta* (formerly *Pezizella discreta*), *Candida ethanolica*, *Brettanomyces anomalus* (formerly *Dekkera anomala*), *Brettanomyces bruxellensis* (formerly *Dekkera bruxellensis*), *Pichia kudriavzevii* (formerly *Issatchenkia orientalis*), *Saccharomyces cerevisiae*, *Hanseniaspora vineae*, and *Ramularia robusta* (formerly *Ilyonectria robusta*). Two Agaricomycete taxa were noted at low abundance (Postia and Melanophyllum); these are not associated with the kombucha fermentation and likely represent contamination from airborne spores.

### Chemical analysis of kombucha

Of the eight organic acids tested for their presence in kombucha, only acetic acid and lactic acid reached detectable levels. Acetic acid was present at 1.06 g/L and lactic acid was present at 0.26 g/L. Additionally, the kombucha contained 11.9 g/L ethanol (1.5% alcohol by volume). Organic acids and ethanol were not detected in the unfermented tea placebo. The placebo pH was 6.30, whereas the kombucha was 3.52. Despite the pH difference, the kombucha had only a slightly more sour taste than the placebo (presumably due to added flavors and sweeteners). Although the kombucha likely contained appreciably less sugar that the placebo (residual sugars were not measured), participants were generally unable to identify which samples they had received.

## Discussion

Despite the considerable popularity of kombucha and the purported health benefits, until very recently, there were virtually no clinical research studies on health benefits in human subjects (13, 41). Indeed, several recent reviews (13, 42–44) found only one study in human subjects (45), and this singular study was neither controlled nor blinded. Only recently has kombucha been used in a controlled human clinical trial (19). In this study, healthy adults (*n* = 11) with normal glucose tolerance, consumed a high glycemic index meal along with one of three beverages - soda water (unsweetened), diet lemonade (sweetened with aspartame and saccharin), or kombucha. Changes in plasma glucose and insulin levels were measured following consumption of this meal and compared to results observed in the same healthy subjects following consumption of a glucose solution. Results showed that kombucha significantly lowered both glycemic and insulin indices compared to consumption of a glucose solution, while neither soda water nor diet lemonade resulted in significant decreases in this measure. The authors suggested that tea components or kombucha microbes may have been responsible for the observed effects (19).

The study reported here, however, is the first clinical trial investigating a health benefit of kombucha among T2D adults in the United States. Our results showed that consumption of kombucha for 4 weeks resulted in significant decreases in fasting blood glucose levels in diabetic subjects with elevated blood glucose levels (>130 mg/dL) compared to baseline, whereas smaller changes in fasting blood glucose observed following consumption of placebo were not significant. This study included two diabetic subjects whose blood sugar was well controlled at baseline (between 80 and 130 mg/dL). In these two subjects, fasting blood glucose increased following kombucha treatment but remained within healthy range (107.4 and 110.3 mg/dL). This pilot study was undertaken, in part, to inform future studies of effect sizes and potential. We observed that fasting blood glucose levels in diabetic patients varied over a wide range (81 to 264 mg/dL) and that some subjects (29%) exhibited fasting blood glucose levels at baseline that were within healthy ranges for diabetics and were unlikely to be further improved by intervention. While the actual reduction in blood glucose when individuals with elevated blood glucose levels at baseline were consuming kombucha was rather large, an average of 74 mg/dL, the variation in baseline levels led to high variation in fasting blood glucose levels after treatment. Assuming such a large reduction occurred in a larger study, a proper sample size for 80 and 90% power calculations would be 18 and 23 participants with elevated baseline blood glucose levels, respectively, or 24 and 30 participants if fasting blood glucose at baseline is unknown. If this outcome was obtained in a well powered study, kombucha could have the potential to greatly affect diabetic care.

The most recent American Diabetic Association consensus statement recommended replacing sugar-sweetened beverages with water as often as possible (46). However, despite the sugar used in the fermentation process of kombucha, daily consumption did not raise fasting blood glucose levels to unhealthy levels in diabetics. This is clinically significant as many diabetics struggle to consume only water and counseling diabetic patients on drink choice is challenging for physicians.

There are several possible explanations for the observed benefit in glycemic control observed in this study. In general, the addition of any carbonated drink to dinner may contribute to appetite suppression and reduced meal size (in both groups), leading to reduced fasting blood glucose. Ginger by itself may have biological activity (47), although the amount consumed was very low (<1%), and ginger was present in both the kombucha and placebo. Additionally, both interventions were effervescent beverages that may have replaced another sugar-sweetened carbonated beverage.

Other possible mechanisms of action may also contribute to glycemic benefits of kombucha. First, as observed in animal models, kombucha was associated with improved pancreatic beta cell regeneration (20, 22) which could contribute to improved endogenous insulin production. Second, chemical constituents formed by kombucha microbes may influence host metabolism and provide health benefits (12, 48–50). These constituents include polyphenols, D-saccharic acid-1,4-lactone, caffeine, organic acids, ethanol, and various alkaloids. Based on *in vitro* and animal studies, several of these compounds have been proposed to prevent oxidative stress-related diseases, such as cardiovascular disorders, cancer, and neurodegeneration, as well as reducing cholesterol levels and blood pressure (41, 50). Third, kombucha has been suggested to contain constituents that reduce starch digestion and reduce net absorbed glucose (51). Kombucha also contains acetic acid, which has been reported to have anti-diabetic properties, although at higher concentrations than the kombucha used in our study (52–54)(several reports). Furthermore, other studies have reported the absence of an anti-glycemic effect (55). Notably, this non-commercial kombucha also contained 1.5% ethanol which, according to several reports (56–59), is unlikely to have affected blood or serum glucose levels. Ultimately, larger studies will be needed to elucidate the exact mechanism of action.

Based on self-reported health outcomes, kombucha was well tolerated, as most individuals reported average or better health measures before and after treatment. Larger studies may have sufficient power to determine if kombucha would impact other health outcomes. For example, previous studies have indicated that the microorganisms present in the kombucha could improve gut health (41, 50, 60), but further studies are needed to investigate this effect. Although the kombucha contained only about 10^6^ organisms per mL, a 240 mL serving would have delivered more than 2 × 10^8^ cells per day. Consistent with previous studies on the kombucha microbiota (60, 61), among the most abundant microbes present in the kombucha were members of the phyla, Bacillota and Pseudomonadota. In particular, the kombucha was dominated by lactic and acetic acid bacteria. Although the relative proportion of lactic acid bacteria and acetic acid bacteria reported for various kombuchas can vary (13, 48, 50, 61, 62), the product used in this study, based on cultural and culture-independent methods, contained nearly equal levels, around 10^6^/mL. Yeasts were also present, including genera and levels typical of kombucha (61).

Ultimately, however, because fecal samples were not obtained, the role of kombucha microbes on the gut microbiota could not be assessed. Nonetheless, to our knowledge, there are no studies showing that kombucha lactic acid bacteria, acetic acid bacteria, or yeasts could contribute to blood glucose lowering, suggesting that the observed effect may be mediated (as noted above) by bioactive end-products produced *in situ* by kombucha microbes.

This study had several limitations. The number of participants was small, and attrition was high. The high rate of attrition was considered to be mainly due to lack of incentive, financial or other, given to participants to complete the entire study. The burden of a daily drink, coordinating deliveries of the drinks, recording daily blood glucose levels, and filling out the questionnaires likely affected subject recruitment. In addition, analyses were dependent on self-reported blood glucose level. Most importantly, we studied only one kombucha. It is possible all kombuchas have similar mechanistic implications but more research into different kombuchas is warranted.

Finally, only one participant reported an adverse event – bloating. Despite the near absence of adverse events among participants, guidelines for safe and hygienic manufacture of kombucha exist to ensure product safety and quality (42).

Kombucha is a growing part of the beverage market in the US and the world (14), driven, in part, by the wide range of suggested health benefits (13). However, nearly all of these benefits are based on *in vitro* or animal studies, and human clinical trials are needed to validate biological outcomes obtained from *in vitro* studies (44).

## Conclusion

In this pilot study, the effect of kombucha consumption on blood glucose levels in adult T2D subjects revealed positive effects. Nonetheless, this study was not sufficiently powered to provide more definitive conclusions. Future studies may help inform physicians when asked about the safety of kombucha by diabetic participants who are concerned about carbohydrate intake.

## Data availability statement

The datasets presented in this study can be found in online repositories. The names of the repository/repositories and accession number(s) can be found at: https://www.ncbi.nlm.nih.gov/, PRJNA928483.

## Ethics statement

This study was approved by the Institutional Review Board committee at MedStar and performed in accordance with their guidance (IRB ID STUDY00001101). All subjects were informed of study design and consented to participate. The patients/participants provided their written informed consent to participate in this study.

## Author contributions

CM, DM, and RH designed the study. CM, SS, DM, VS, and SD participated in subject recruitment. CC, CK, HH-A, and RH performed kombucha microbiota and chemical analyzes. CM, SS, DM, CC, VS, SD, JA, CK, HH-A, and RH analyzed data. CM, CC, CK, and JA generated figures. CM, DM, and RH wrote the manuscript. CC, CK, HH-A, and JA edited the manuscript. All authors contributed to the article and approved the submitted version.

## Conflict of interest

RH is a co-founder of Synbiotic Health; JA has a financial interest in Synbiotic Health. DM serves as President of the Board of Directors of the International Scientific Association for Probiotics and Prebiotics, a non-paid position. All kombucha and placebo drinks were donated by Craft Kombucha. Craft Kombucha did not have any access to data reported in this study. No author has any financial ties with Craft Kombucha. SD was employed by MedStar Health.

The remaining authors declare that the research was conducted in the absence of any commercial or financial relationships that could be construed as a potential conflict of interest.

## Publisher’s note

All claims expressed in this article are solely those of the authors and do not necessarily represent those of their affiliated organizations, or those of the publisher, the editors and the reviewers. Any product that may be evaluated in this article, or claim that may be made by its manufacturer, is not guaranteed or endorsed by the publisher.
