# Supplementary material for: Kombucha tea as an anti-hyperglycemic agent in humans with diabetes – a randomized controlled pilot investigation
Source: Front Nutr. 2023 Aug 1;10:1190248. doi: 10.3389/fnut.2023.1190248 (PMC10426908; doi:10.3389/fnut.2023.1190248)
Supplement: Supplementary file 1 [file Data_Sheet_1.PDF]

Supplement File 1

Kombucha Research Participant Health Questionnaire

Timing of questionnaire (circle one):

Before first drink / 1 week of drinking first drink / 4 weeks of drinking first drink Before second drink / 1 week of drinking second drink / 4 weeks of drinking second drink

1. Overall health: How would you rate your overall health?

Worst ever / worse than usual / average / better than usual / best ever

2. Blood sugar control

Over the past week, what was your blood sugar readings? (best if you recorded before breakfast)

Saturday _______

Sunday ________

Monday _______

Tuesday ________

Wednesday _______

Thursday ________

Friday ________

How many episodes of hypoglycemia (low blood sugar) have you experienced in the past week

(i.e., blood sugars less than 65)? _______

If you take insulin, has your insulin requirement changed in the past week?

Increased / No change / Decreased

3. Gut Health:

How would you rate your overall gut health?

Worst ever / worse than usual / average / better than usual / best ever

How many days a week do you feel bloated or gassy?

Not at all / Several days / More than half the days / Nearly every day

How many days a week do you feel constipated or had hard stools?

Not at all / Several days / More than half the days / Nearly every day

How many days a week do you have diarrhea or loose stools?

Not at all / Several days / More than half the days / Nearly every day

How many days a week are you concerned how long you spend on the toilet?

Not at all / Several days / More than half the days / Nearly every day

4. Skin Health

How would you rate your overall skin health?

Worst ever / worse than usual / average / better than usual / best ever

How often do you experience skin problems such as dry skin, eczema, rashes, or acne?

Not at all / Several days / More than half the days / Nearly every day

5. Anxiety and depression: How often have you been bothered by the following problems?

Feeling nervous, anxious or on edge

Not at all / Several days / More than half the days / Nearly every day

Not being able to stop or control worrying

Not at all / Several days / More than half the days / Nearly every day

Little interest or pleasure in doing things

Not at all / Several days / More than half the days / Nearly every day

Feeling down, depressed or hopeless

Not at all / Several days / More than half the days / Nearly every day

6. Blood Pressure

Over the past week, what was the range of your blood pressure readings?

____

7. General (at the end of 4 weeks)

Would you recommend this drink to a friend? Yes / No / unsure

How many days this week did you drink at least 6 ounces of the research drink? ____

Which drink do you think you were drinking? Kombucha / placebo / unsure

8. Vulvovaginal health (women only)

How would you rate your overall vulvovaginal health?

Worst ever / worse than usual / average / better than usual / best ever

Does your vulva or vagina itch, burn, or sting?

Not at all / Several days / More than half the days / Nearly every day

Do you have discharge or odor from your vulva or vagina?

Not at all / Several days / More than half the days / Nearly every day

How often do you get vaginal bacterial or yeast infections?

Never / Rarely / Occasionally / Often / All the time
