# Supplementary material for: Kombucha tea as an anti-hyperglycemic agent in humans with diabetes – a randomized controlled pilot investigation
Source: Front Nutr. 2023 Aug 1;10:1190248. doi: 10.3389/fnut.2023.1190248 (PMC10426908; doi:10.3389/fnut.2023.1190248)
Supplement: Supplementary file 2 [file Data_Sheet_2.PDF]

Supplement File 2. Health questionnaire responses: perceived health.

			Worst ever	Worse than usual	Average	Better than usual	Best ever
Overall health	Kombucha	baseline	1	2	3	3	
		Week 1	1		6	1	
		Week 4	1	1	1	1	1
	Placebo	baseline		3	6	1	
		Week 1	1		4	1	1
		Week 4	1		5	1	
Gut health	Kombucha	baseline		1	7	1	
		Week 1		1	4	2	1
		Week 4			1	2	2
	Placebo	baseline	1	1	7		1
		Week 1		1	3	1	2
		Week 4			5	1	1
Vulvovaginal health	Kombucha	baseline			1	4	2
		Week 1			3	1	2
		Week 4				2	2
	Placebo	baseline	1	1	4	1	1
		Week 1		1	4	1	
		Week 4			3	1	2

Skin health	Kombucha	baseline		3	3	3	
		Week 1	1		4	2	1
		Week 4	1		2	1	1
	Placebo	baseline		2	7		1
		Week 1			5	1	1
		Week 4			5	2	
