# Supplementary material for: Kombucha tea as an anti-hyperglycemic agent in humans with diabetes – a randomized controlled pilot investigation
Source: Front Nutr. 2023 Aug 1;10:1190248. doi: 10.3389/fnut.2023.1190248 (PMC10426908; doi:10.3389/fnut.2023.1190248)
Supplement: Supplementary file 3 [file Data_Sheet_3.PDF]

Supplement File 3. Health questionnaire responses: symptoms frequency.

			Not at all	Several days	More than half the days	Nearly every day
Gut health	Kombucha	baseline	25	5	4	2
		Week 1	19	9	2	2
		Week 4	14	3	0	3
	Placebo	baseline	23	9	1	6
		Week 1	15	7	2	4
		Week 4	16	9	2	1
Vulvovaginal health	Kombucha	baseline	15	4	2	0
		Week 1	17	0	1	0
		Week 4	11	0	1	0
	Placebo	baseline	18	3	2	1
		Week 1	13	4	1	0
		Week 4	14	3	1	0
Skin health	Kombucha	baseline	2	2	3	2
		Week 1	2	3	1	2
		Week 4	2	1	0	2
	Placebo	baseline	0	5	3	2
		Week 1	2	3	1	1

		Week 4	2	3	0	2
Mental health	Kombucha	baseline	19	14	3	0
